# New insights on the potential effect of orexin receptor antagonist suvorexant on Parkinson’s disease symptoms

**DOI:** 10.1007/s10072-023-07261-2

**Published:** 2024-01-17

**Authors:** Hayder M. Al-kuraishy, Ali I. Al-Gareeb, Athanasios Alexiou, Marios Papadakis, Hebatallah M. Saad, Gaber El-Saber Batiha

**Affiliations:** 1 Department of Clinical Pharmacology and Medicine, College of Medicine, Al-Mustansiriyia University, P.O. Box 14132, Baghdad, Iraq; 2 Department of Science and Engineering, Novel Global Community Educational Foundation, Hebersham, NSW 2770 Australia; 3 Department of Research & Development, AFNP Med, Wien, 1030 Austria; 4 University Centre for Research & Development, Chandigarh University, Chandigarh-Ludhiana Highway, Mohali, Punjab India; 5 Department of Research & Development, Funogen, Athens, Greece; 6 Department of Surgery II, University Hospital Witten-Herdecke, University of Witten-Herdecke, Heusnerstrasse 40, 42283 Wuppertal, Germany; 7 Department of Pathology, Faculty of Veterinary Medicine, Matrouh University, Matrouh, Matrouh, 51744 Egypt; 8 Department of Pharmacology and Therapeutics, Faculty of Veterinary Medicine, Damanhour University, Damanhour, 22511 AlBeheira Egypt

Dear editor,

The Holy Quran mentioned “And it is He who made the night as clothing for you, and sleeps as a rest, and made the day a resurrection.” Orexin also known as hypocretin is a neuropeptide released from the perifornical area of the lateral hypothalamus involved in wakefulness, regulation of sleep, neurogenesis, cognition, and energy homeostasis [1]. It was discovered in 1988 and shown to act on orexin receptors which are expressed peripherally and centrally. There are two types of orexins: orexin-A acts on both orexin-1 and orexin-2 receptors, while orexin-B acts mainly on orexin-2 receptors [1]. Dysregulation of the orexin pathway is associated with the development and progression of neurodegenerative diseases (NDDs) including Parkinson’s disease (PD). PD is a progressive NDD characterized by the degeneration of dopaminergic neurons in the substantia nigra due to the deposition of α-synuclein and linked inflammatory and oxidative stress disorders. Orexin has a neuroprotective role against PD neuropathology according to preclinical and clinical studies [2]. Orexin dysfunction promotes the progression of PD neuropathology by enhancing the aggregation of α-synuclein which in turn triggers the degeneration of dopaminergic neurons [2, 3]. Clinical presentation of PD includes motor symptoms like rigidity, resting tremor, bradykinesia, and postural instability, and non-motor symptoms include cognitive impairment, autonomic dysfunction, depression, and sleep disorders. Notably, non-motor symptoms precede the development of motor symptoms over many years. In the early and intermediated phase of PD, neuropathology orexin level is increased as a compensatory mechanism to mitigate inflammatory and oxidative stress disorders in PD [2]. A case–control study conducted by Huang et al. [2] revealed that orexin plasma level was increased in the early and intermediated phases of PD patients compared with healthy controls. High orexin plasma level was correlated with the severity of non-motor symptoms mainly REM sleep disorders [2]. It has been shown that restoration of sleep patterns prevents the progression of PD neuropathology, as insomnia increases aggregation and attenuates clearance of α-synuclein. In this bargain, orexin receptor antagonists could be effective in the management of sleep disorders and non-motor symptoms in PD patients. Suvorexant is a dual orexin-1 and orexin-2 receptor antagonist orally active medication approved in 2014 for the management of sleep disorders including insomnia [4]. Unlike, benzodiazepines, it increases REM sleep duration with minimal effect on NREM sleep. Therefore, theoretically, suvorexant could be effective in the management of sleep disorders in PD patients. However, in 2017, a case reported by Tabata et al. [5] showed that suvorexant use for the management of insomnia in a 72-year-old male PD patient led to severe delirium and nightmares resembling REM sleep behavior disorder (RBD) which is a common non-motor symptom in PD. To our knowledge, there is no clinical study evaluating the effect of suvorexant on motor and non-motor symptoms in PD patients.

Taken together, orexin is a neuroprotective neuropeptide that prevents the degeneration of dopaminergic neurons and the development of PD. Inhibition of the orexin signaling pathway by suvorexant and other orexin receptor antagonists may exacerbate the progression of both motor and non-motor symptoms in PD. This letter prompts for a clinical trial to evaluate the clinical profile of suvorexant in PD and also to estimate the clinical efficacy of orexin receptor agonists against the development and progression of PD. From these recommendations, we can know why David takes on Goliath.

## Data Availability

Data sharing is not applicable to this article as no data sets were generated or analyzed during the current study.
